# Trade-Off between Bile Resistance and Nutritional Competence Drives *Escherichia coli* Diversification in the Mouse Gut

**DOI:** 10.1371/journal.pgen.1002107

**Published:** 2011-06-16

**Authors:** Marianne De Paepe, Valérie Gaboriau-Routhiau, Dominique Rainteau, Sabine Rakotobe, François Taddei, Nadine Cerf-Bensussan

**Affiliations:** 1 INSERM, U989, Université Paris Descartes, Paris, France; 2 INRA, UMR1319 Micalis, Domaine de Vilvert, Jouy-en-Josas, France; 3 INSERM, ERL U1057/UMR7203, Université Pierre et Marie Curie, Paris, France; 4 INSERM, U1001, Université Paris Descartes, Paris, France; Universidad de Sevilla, Spain

## Abstract

Bacterial diversification is often observed, but underlying mechanisms are difficult to disentangle and remain generally unknown. Moreover, controlled diversification experiments in ecologically relevant environments are lacking. We studied bacterial diversification in the mammalian gut, one of the most complex bacterial environments, where usually hundreds of species and thousands of bacterial strains stably coexist. Herein we show rapid genetic diversification of an *Escherichia coli* strain upon colonisation of previously germ-free mice. In addition to the previously described mutations in the EnvZ/OmpR operon, we describe the rapid and systematic selection of mutations in the flagellar *flhDC* operon and in *malT*, the transcriptional activator of the maltose regulon. Moreover, within each mouse, the three mutant types coexisted at different levels after one month of colonisation. By combining *in vivo* studies and determination of the fitness advantages of the selected mutations in controlled *in vitro* experiments, we provide evidence that the selective forces that drive *E. coli* diversification in the mouse gut are the presence of bile salts and competition for nutrients. Altogether our results indicate that a trade-off between stress resistance and nutritional competence generates sympatric diversification of the gut microbiota. These results illustrate how experimental evolution in natural environments enables identification of both the selective pressures that organisms face in their natural environment and the diversification mechanisms.

## Author Summary

The mechanisms generating the huge biodiversity on earth are not entirely understood. Bacterial populations are powerful models to explore the mechanisms of evolution, owing to their big population size, rapid growth, and high mutation rate. One of the more complex bacterial community is the mammalian gut microbiota, and *Escherichia coli* is one of the first colonizers of the newborn intestine. Herein we studied diversification of an *Escherichia coli* strain in germ-free mice, a simplified though ecologically relevant system. We show rapid genetic diversification upon colonization, characterized by the systematic selection of mutations in three different pathways: in the global regulator EnvZ/OmpR controlling outer membrane permeability, in the flagellar operon, and in the maltose regulon. By combining *in vivo* and *in vitro* experiments, we show that the selective forces that drive *E. coli* diversification are the presence of bile salts and the competition for nutrients. Altogether our results indicate that a trade-off between stress resistance and nutritional competence is sufficient to mediate diversification of bacteria. These results illustrate how experimental evolution in natural environments allows the identification of the selective pressures that organisms face in their natural environment, as well as the diversification mechanisms.

## Introduction

Understanding why there are so many species is a fundamental problem in evolutionary biology that is far from being understood. Numerous experimental evolution studies have shown that the amount of diversity that evolves from initially genetically uniform population increases as a function of environmental complexity, in terms of spatial heterogeneity [1], temporal variability, or types of food resource [2], but can also happen in a constant environment [3]. However, to our knowledge, very few experimental evolution studies addressing the question of diversification have been carried out in natural environments [4]. These type of experiments are however very useful to identify the mechanisms that operate on diversification in complex environments.

Bacterial populations are powerful models to explore the mechanisms of evolution, in particular because they allow the connection of phenotype to genotype. The distal part of the mammalian gut is one of the most densely populated microbial ecosystems on earth with more than 10^11^ organisms per milliliter of luminal content. The gut microbiota community belongs to a limited number of phyla but comprises hundreds of species and thousands of strains [5], [6], [7], whose coexistence is made possible by a partitioning of substrate utilization [8], [9], [10]. Diversity can also be achieved when the product of one microbe metabolism becomes the substrate for another [11]. However, the mechanisms underlying the coexistence in the gut of numerous closely related species or even strains that possess the same metabolic capabilities remain elusive. This is notably the case for the enterobacteria *Escherichia coli*, as several strains usually coexist in the digestive tract [12], [13], [14].

The gastrointestinal tract of germ-free mice enables the study of reciprocal mechanisms of adaptation between bacteria and their hosts, as it represents a simplified and controlled gut environment. In the case of *E. coli* colonization, it moreover offers an ecologically relevant environment model, as *E. coli* is one of the first colonizers of the mammalian newborn germ-free intestine [12]. We have previously shown that, following colonization of germ-free mouse gut by the *E. coli* MG1655 strain, bacteria possessing point mutations in the *ompB* operon were rapidly selected and reached 90% of the total population four days after inoculation [15]. The *ompB* product is the EnvZ/OmpR signal transduction system, the global regulator of adaptation to changes in osmolarity. We demonstrated that the fitness gain provided by these mutations *in vivo* results mainly from two distinct effects on flagellar expression and membrane permeability, both reduced in the selected *ompB* mutants [15].

Herein, we show that, although *ompB* mutants initially possess a very high selective advantage, they never get fixed in the bacterial gut population. Rather, bacteria with mutations either in the *malT* gene or in the *flhDC* operon are repeatedly and concomitantly selected in the minority wild type *ompB* population. We present *in vivo* and *in vitro* evidence that this diversification is driven by a trade-off between resistance to bile salts and nutritional competence.

## Results

### Rapid and systematic diversification in the mouse gut

#### Recurrent selection of three different morphotypes in gnotobiotic mice

**Figure 1:**
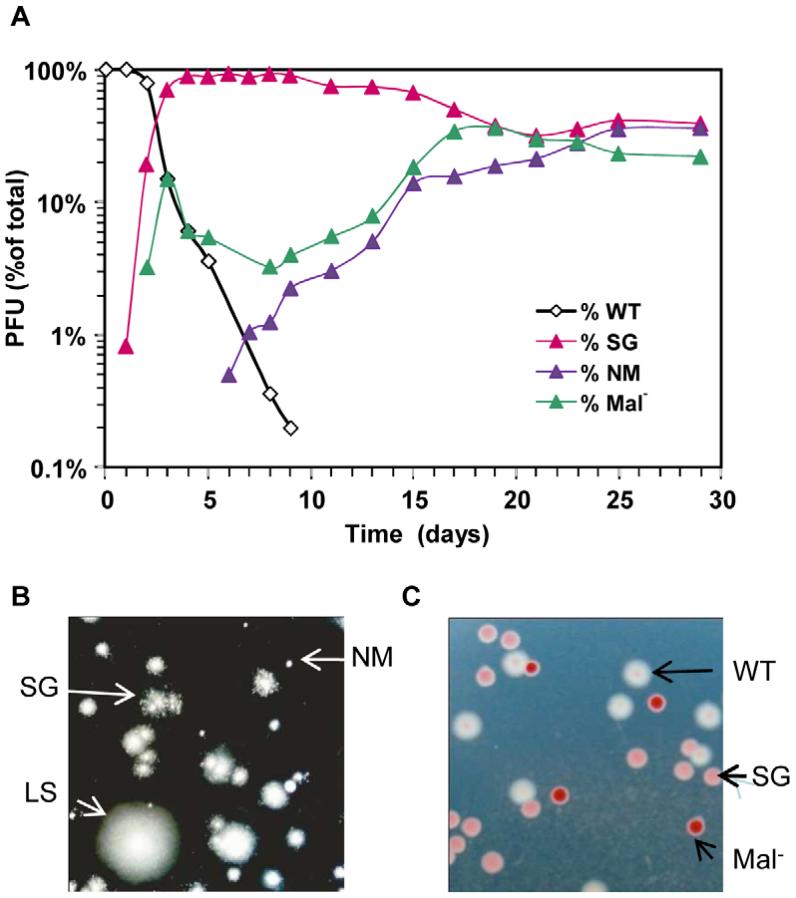
Rapid diversification occurs during gut colonization. (A) Evolution over time (in days) of CFU morphotypes in the feces of one representative mouse (out of 8 studied) inoculated with *E. coli*. (B) Phenotypes of bacteria from the same feces observed in motility agar: large smooth (LS, similar to the ancestor), small granulous (SG) and totally non motile (NM). (C) Colonies observed on tetrazolium maltose plates. White colonies correspond to the ancestral Maltose+ phenotype, pink colonies correspond to SG colonies that display a reduced ability to use maltose, and red colonies are unable to use maltose (Mal-).

In keeping with our previous results [15], *ompB* mutants forming small granular (SG) colonies on motility plates were very efficiently selected during the first two days following colonization. The selective advantage of these mutants however decreased rapidly (Figure 1A and Figure S1). The change of fitness of *ompB* mutants was independent of their frequency in the bacterial population (Figure S1) and might thus result either from the selection of distinct mutations in the population carrying the ancestral allele of *ompB,* and/or from changes in the gut environment following colonization. In agreement with the first hypothesis, we observed the selection of two other distinct bacterial morphotypes (Figure 1B).

After three days of colonization, only 20% of bacteria still exhibited the ancestral motility phenotype. This population was however not homogenous, as 40% of bacteria had lost the ability to use maltose (Mal^−^ phenotype), as revealed by their red colour on agar plates containing maltose sugar and tetrazolium dye (maltose tetrazolium plates). In every mouse, by day 10, all motile colonies tested displayed a Mal^−^ phenotype on tetrazolium plates. Despite their very early selection, these maltose deficient bacteria did not invade the population, and their proportion remained between 10 and 20% after 30 days of colonization (Figure 1A). All Mal^−^ colonies tested were motile like the ancestor, suggesting that the Mal^−^ phenotype is never or rarely selected in the *ompB* mutants. Moreover, sequencing the *ompB* operon in four Mal^−^ colonies confirmed the absence of mutation. Interestingly, the maltose regulon expression is reduced in *ompB* mutants, as revealed by the pink color of their colonies on maltose tetrazolium plates (Figure 1C). This result was confirmed by RT-qPCR on the *lamB* gene of the maltose regulon (Figure S2), and is consistent with previous descriptions of *envZ* point mutations that reduce maltose regulon expression [16], [17]. This reduced expression of maltose genes in *ompB* mutants may circumvent the need for complementary adaptive mutation in the maltose regulon.

In addition to Mal^−^ colonies, bacteria forming completely non-motile (NM) colonies were first observed after 5 to 15 days of colonisation. NM colonies proportion rapidly increased and stabilized after days 15–20 at various levels in each of the different mice (Figure 1A and Figure S3 for individual mouse). Interestingly, the NM phenotype was selected almost exclusively in the bacterial population displaying the maltose ancestral phenotype (in 7 out of 8 mice). Moreover, none of the 5 sequenced NM clones possessed a mutation in the *ompB* operon. Since the population with ancestral motility and maltose phenotypes represented less than 1% of the total bacteria when NM mutants were selected, this observation suggested strong counter selection of the NM phenotype in *ompB* mutants and in Mal^−^ bacteria.

#### Identification of the mutations leading to the Mal^−^ and NM phenotypes

**Figure 2:**
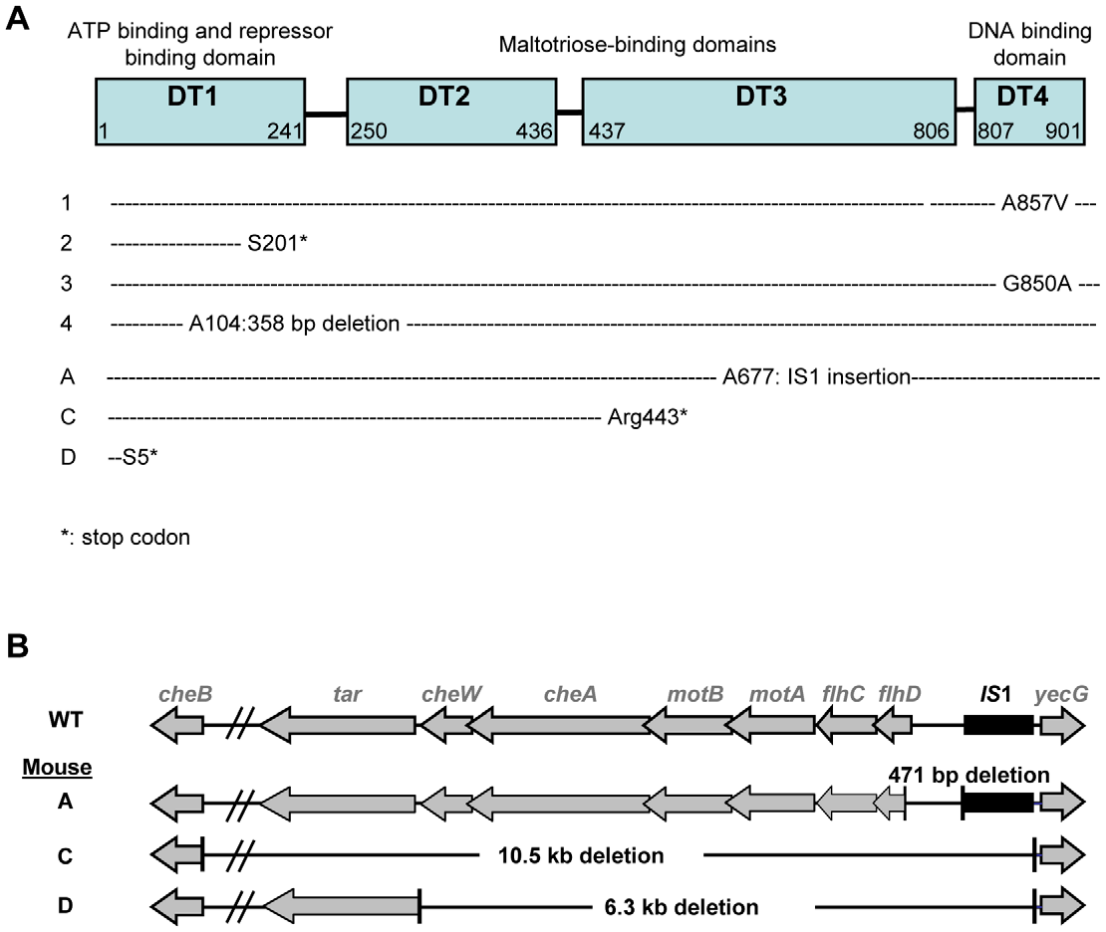
Identification of selected mutations. (A) Mutations in *malT* gene were analyzed in 7 Mal- clones, each isolated from a different mice. Different mutations were selected, that all led to a null phenotype. (B) Genetic map of the deletions detected in three non-motile clones isolated from independent mice. All non-motile clones tested possessed a deletion in the region immediately downstream of *flhDC*.

Maltose deficient bacteria remained capable of using other simple sugars (Table 1), suggesting that mutations conferring the Mal^−^ phenotype affected only the maltose regulon. As the maltose porin *lamB*, encoded in the maltose regulon, is also the receptor for bacteriophage lambda, we tested lambda sensitivity of eight Mal^−^ clones, each isolated from a different mouse 5 days after colonization. All clones were resistant to lambda infection, pointing to mutations in *malT* gene, the transcriptional activator of the maltose regulon [18]. Sequencing of the *malT* genes from each clone confirmed that each of them possessed an inactivating mutation (Figure 2A). Transformation of the Mal^−^ bacteria with a plasmid possessing the *malT* gene under its own promoter restored the ability to use maltose.

**Table 1:** Maximal growth rates of the ancestral strain (WT) and isogenic mutants in minimal media containing a single source of energy.

	Minimal doubling time (minutes) *			
	WT	*ompB* ^SG1^	Δ*flhDC*	Δ*malT*
**D-glucose**	42.8±1.3	66.9±1.5	51.5±3.1	44.8±2.1
**N-acetyl G^$^**	47.8±2.1	78.2±4.3	52.6±2.7	46.9±1.4
**L-arabinose**	57.7±1.3	93.4±1.1	69.6±1.5	53.3±1.7
**L-fucose**	61.3±2.5	99.6±2.2	68.3±3.9	61.6±2.1
**D-galactose**	93.2±2.7	98.9±1.3	137.9±8.9	107.5±1.0
**D-gluconate**	46.3±3.0	75.8±3.0	61.0±4.2	52.5±5.4
**D-glucuronate**	56.6±5.4	83.5±4.0	71.4±8.3	58.9±2.4
**Lactose**	41.6±4.5	84.0±1.8	nd	44.1±1.6
**Maltose**	44.4±0.3	93.2±0.5	48.1±0.6	∞
**D-mannose**	71.2±2.1	108.4±5.1	79.6±3.4	96.5±9.9
**Xylose**	89.1±4.3	115.9±0.4	84.7±0.8	91.8±3.7

*mean +/− s.e.m. on three to four independent measurements.

$ N-acetyl-D-glucosamine.

nd: not done.

The growth rate is indicated by the maximal doubling time of the strain in M9 media containing the specified sugar.

Identification of the mutations conferring the NM phenotype was guided by our previous results in gnotobiotic mice colonized with an *E. coli* strain deleted for the *ompF* gene, encoding the major outer membrane porin OmpF [15]. In this work, we have observed a rapid selection of non-motile mutants bearing genomic deletions in the downstream region of *flhDC* operon, encoding the master regulator of flagellum biosynthesis, FlhD(2)C(2). Deletions in the same genomic region were observed by PCR in five NM mutants isolated from three different mice (Figure 2B). Complementation with a plasmid containing the *flhDC* operon restored the motility phenotype for the three mutants isolated from mouse A that had a deletion limited to *flhDC* operon. PCR analysis showed that the three mutants had the same deletion sizes, suggesting that in each mouse, the majority of the *flhDC* (NM) mutants are clonal descendants of the same mutant. That *flhDC* mutants were not secondarily selected from *ompB* mutants may be explained by the strongly decreased *flhDC* expression in *ompB* mutants. Therefore, the gain of fitness conferred by the total loss of expression would be probably minimal. The rare selection of *flhDC* mutants in the *malT* population (observed in only 1 out of 8 studied mice, data not shown) is however intriguing and suggests a negative interaction between these two mutations.

The independent systematic and rapid selection of mutations in the same three genes under identical experimental conditions is evidence for a strong selective advantage during gut colonization [19]. Moreover, the adaptive radiation observed suggests niche specialization. Indeed, stable coexistence of genotypes is strongly favoured by spatial partitioning and/or metabolic specialization and concomitant fitness trade-offs [20]. The mechanisms driving the diversification of bacteria within the gut were further addressed by combining *in vivo* and *in vitro* experiments to define the factors conditioning the selection of the mutants and enabling their coexistence.

### *In vivo* investigation of host factors that could influence diversification

#### Role of the host innate immune response

Host immune responses have a strong influence on the composition of the gut microbiota composition [21] and microbial evolution. Notably, immunoselection of bacterial epitopes can drive the evolution of bacterial cell surface components [22]. Moreover intestinal inflammation can profoundly alter the species distribution of the microbiota, presumably by eliminating species highly sensitive to bactericidal factors released by the inflamed gut [23] and/or changing the competition for resources [24]. As a result of flagellin (FliC) down-regulation, *ompB* mutants lack the pro-inflammatory properties of the ancestral strain [15] and may be selected by their avoidance of immune responses. To test this hypothesis, we used mice deficient for the protein MyD88, which are unable to transduce proinflammatory signals upon flagellin binding to Toll-like receptor 5 (TLR5), its cognate receptor on epithelial cells [25], [26]. MyD88−/− mice were first treated for three weeks by a cocktail of large spectrum antibiotics to sterilize the intestine and subsequently colonized with the *E. coli* MG1655 strain. Bacterial diversification and early selection of *ompB* mutants were comparable to those observed in WT mice (Figure S4). Moreover, despite the *in vitro* pro-inflammatory property of the MG1655 strain [26], no inflammatory response could be detected in the intestines of WT mice following colonization (Figure S5 and [27]), a result in keeping with another recent study [28]. Taken together, these results strongly argue against a role of the innate immune response in the diversification of *E. coli* during gut colonization as seen here, in particular in the selection of mutants with reduced flagellar expression.

#### Role of spatial distribution

In *E. coli*, flagella are indispensable for chemotaxis and thus for fitness in structured environments presenting a gradient of nutrients [29]. Yet important variability in motility has been observed among *E. coli* natural isolates, with about 50% of commensal and pathogenic isolates being non-motile or very poorly motile, suggesting that the loss of motility is often selected for [30]. Motility has been associated with virulence in some pathovars [31], [32], or on the contrary with the loss of virulence for other pathovars [33]. Moreover, in the case of commensal strains the role of motility in the gut is still not understood. It has been suggested that motility enables bacteria to colonize the mucus layer that covers epithelial cells [34]. Indeed numerous sugars in the mucus are metabolic substrates for *E. coli* and motility might be selected despite the potential costs associated with flagella expression. Spatial distribution of bacteria was analyzed after 20 days of colonization by confocal microscopy in mouse colon and caecum as these two gut compartments harboured the highest density of *E. coli* populations. Motile bacteria were distinguished from Δ*flhDC* and *ompB* mutants by their expression of yellow fluorescent protein (YFP) as reporter of FliC (flagellin) production [15]. The proportion of flagellated bacteria expressing FliC was comparable in the caecum and the colon and did not change with the distance from the epithelial surface (Figure S6), suggesting that flagellar expression does not improve mucus colonization ability.

To gain further insight into the cause of diversification, we investigated *in vitro* how gut environmental characteristics might promote the selection process.

### *In vitro* investigation of fitness gains conferred by the selected mutations

A well described source of diversification is resources specialization, provided that trade-offs exist in the ability to exploit alternative resources [35], [36]. This mechanism is attractive in our case, since the gut contains a wide diversity of nutrients. Growth modifications conferred by the selected mutations were therefore investigated in controlled media containing a single energy source.

#### Growth on minimal medium supplemented with simple sugars

**Figure 3:**
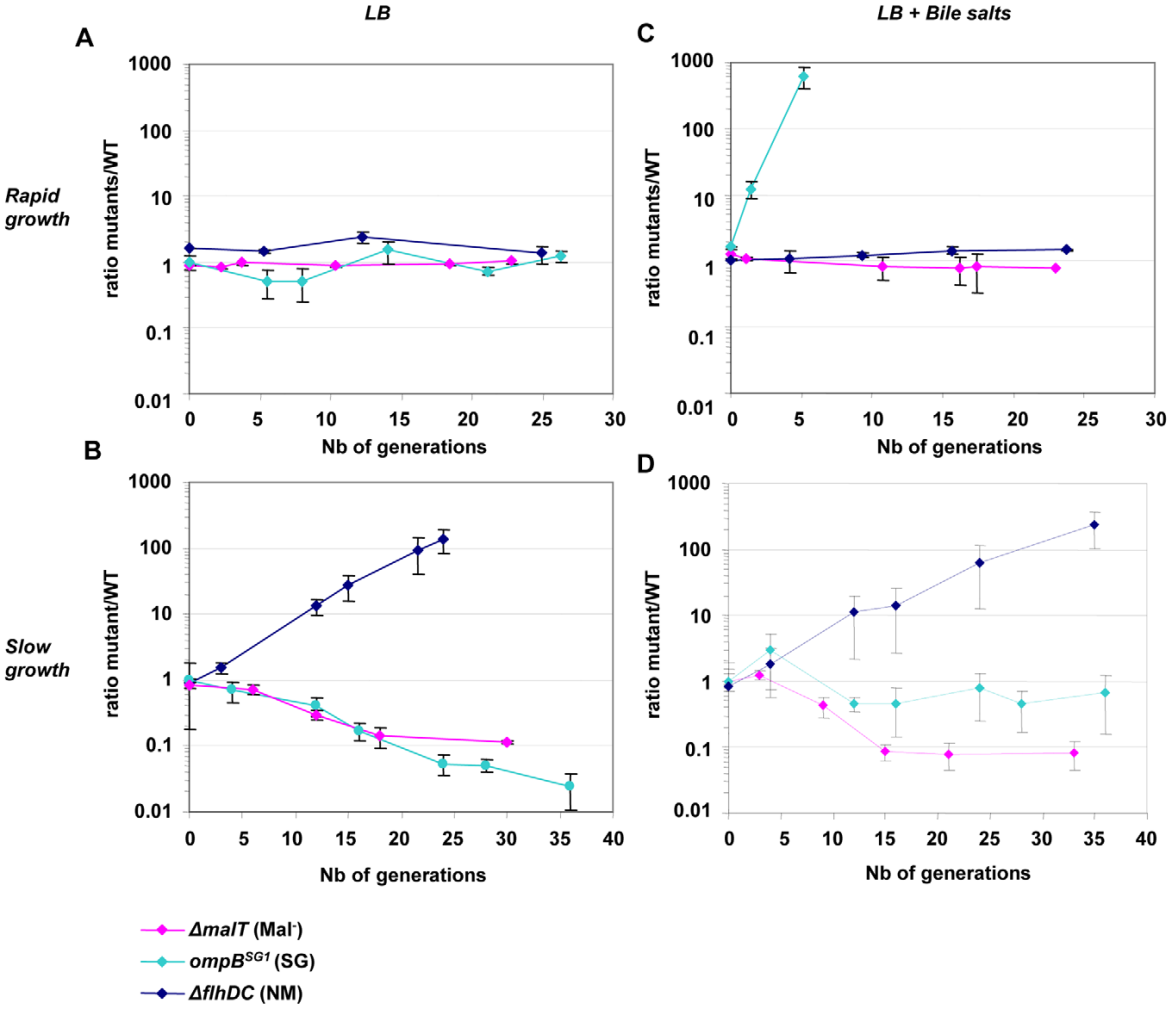
Deletion of *flhDC* genes confers a growth advantage in chemostat. Evolution over time (in days) of the ratio of mutant (green) to WT (red) CFU in chemostats inoculated with E. coli MG1655 and either isogenic *ompB* ^SG1^ (possessing the point mutation in *ompB* of the SG1 mutant, pale blue symbols), ▵*flhDC* (dark blue symbols) or ▵*malT* (pink symbols) strain mixed at initial ratios of 1∶1. (A) Competition in rich LB media (doubling time of 20 minutes). (B) Competition in chemostat, in condition of limited nutrient availability (doubling time of 160 minutes). (C) Competition in rich LB media but in the presence of bile salts at 0.8% w/v. (D) Competition in limited nutrient availability and in the presence of bile salts. After one day (about ten generations), NM mutants appear in the WT population, likely accounting for the change in the outcome of the competition. Means and standard errors of four experiments are shown.

It has been suggested that Δ*flhDC* MG1655 mutants are selected in the gut owing to their modified expression of genes involved in the Entner-Doudoroff pathway and a resulting advantage in the use of simple sugars [37], [38]. To investigate an eventual nutritional specialization of the selected mutants, we constructed isogenic mutants by introducing the mutation from the *ompB* mutant SG1, by deleting *flhDC* or by deleting *malT* in WT strains distinguishable by fluorescent reporter genes. All *in vitro* experiments presented below have been done with these isogenic strains.The growth rates of the resulting *ompB^SG1^*, Δ*flhDC* and Δ*malT* strains were compared on minimal M9 medium supplemented with one of 11 different sugars, including those present in intestinal mucus [39]. None of the deletions increased the maximal growth rate (Table 1), thus excluding the possibility that the mutants were selected for their improved maximal growth rate on simple sugars. On the contrary, this experiment revealed that the *ompB^SG1^* mutation resulted in a significant decrease of maximal growth rate on all the sugars, and that Δ*flhDC* and Δ*malT* mutants were also growing slowly on some of these sugars (L-arabinose, D-galactose and D-mannose). OmpR controls the expression of a large number of genes involved in several pathways, mainly involved in outer membrane permeability but also in metabolism [40]. The reduced growth rate observed in minimal medium might be linked with the reduced outer membrane permeability of the strain, crucial for nutritional competence. Accordingly *ompB* mutation did not modify the strain fitness during exponential growth in rich LB medium (Figure 3A). The slower growth of the Δ*flhDC* strain on galactose might be due to FlhD(2)C(2) control of the transporter of this sugar. A decreased growth rate on gluconate and glucuronate was also expected, as *flhDC* controls the expression of *edd* gene. Indeed, *edd* stimulates the Entner-Doudoroff pathway, which participates in the metabolism of the latter sugars [41], [42]. However, we do not have any explanation for the slower growth on other sugars. The phenotype of the Δ*malT* strain might result from the role of porin LamB in the transport at low concentrations of simple sugars, such as glucose and galactose [43].

#### Growth in nutrient-limited conditions

In the aforementioned growth experiments in defined media, we noticed that, in some conditions, the *flhDC* deletion increased optical density of cultures grown overnight by about 10% (Figure S7), a result consistent with previously published studies [44], [45]. As the maximal culture density can depend on growth capacity in nutrient and oxygen-limited conditions, we grew the different strains in anaerobic continuous cultures (chemostats). These conditions mimic those in the gut lumen, where bacteria grow under very low oxygen pressure [46] and under nutrient restriction due to high bacterial density. Cultures were grown in LB medium, in order to avoid limitation of a specific component, which is not expected in the gut environment. We set the doubling time of bacteria at two hours. This time lapse is much longer than the twenty minutes observed in batch conditions, and is close to the growth rate estimated in mouse intestine (between 80 and 120 minutes Anaerobiosis in the cultures was monitored by transcriptional activation of genes regulated by oxygen availability (Figure S8). In these conditions, we observed that the *flhDC* mutant, and to a lesser extent the *ompB^SG1^* mutant, reached much higher cell density than the WT strain (Table 2).

**Table 2:** Bacterial density in chemostats.

Strain	Bacterial density (0D 600 nm)
**WT**	0.408±0.021
***ompB^SG1^***	0.570±0.038
***▵malT***	0.398±0.027
***▵flhDC***	0.671±0.017
***▵rpoS***	0.405±0.025
***▵rpoS ▵flhDC***	0.686±0.034

Optical density of bacterial cultures in chemostats at equilibrium (after 24 hours of growth). The *flhDC* mutant, and to a lesser extent the *ompB^SG1^* mutant, reach a higher density.

Since RpoS is the master regulator of the stationary stress response and negatively regulates genes necessary for growth under starvation, we tested the impact of deleting *rpoS* gene in WT and Δ*flhDC* strains on the final bacterial density in chemostats. Bacterial density was not affected by rpoS deletion, precluding a role of the general stress response in the phenotype of *flhDC* mutants in chemostats (Table 2).

In order to measure more precisely the advantage of the selected mutations, competition experiments were performed in chemostats between the ancestor and the mutants selected *in vivo.* Strains were distinguished by their motility, by inducible fluorescent markers, and by their ability to use maltose. In chemostats, the *flhDC* deletion conferred a dramatic gain of fitness as the Δ*flhD* strain outcompeted the WT strain by 100 fold within two days, which corresponds to a selective advantage of 18%. The *ompB^SG1^* and Δ*malT* strain were however slightly disadvantaged (Figure 3B).

In order to understand the advantage of the *flhDC* mutant under conditions of limited nutrient availability, *fliC* expression was assessed by measuring YFP fluorescence of the WT *pfliC-yfp* strain in various growth conditions (Table 3). FliC expression was approximately tenfold higher in chemostat and in the gut than during exponential growth in rich LB medium. During early exponential growth in LB, the cost of flagella synthesis has been evaluated to represent over 2% of total energy expenditure [47]. The cost of flagella expression in the gut or in chemostats could thus represent as much as 10% to 20% of the total energy expenditure. This value is in the same order of magnitude as the selective advantage of the *flhDC* strain measured in chemostat.

**Table 3:** High flagella expression in the gut and in chemostat.

	Relative flagella expression (p*fliC*/*rrnB* P2 ratio)
**Rapid growth (exponential phase)**	0.17±0,03
**Stationary phase**	0.56±0,01
**Slow growth (chemostat)**	1.80±0,02
**Mice (d1 to d4)**	1.94±0,15

The relative expression of FliC, the main component of the flagella, is determined by the ratio of YFP fluorescence (due to p*fliC-yfp* construct expression) to CFP fluorescence (due to the rrnB P2-cfp expression). Mean ratio of YFP to CFP fluorescence +/− the s.e.m. of three to nine (in the case of mice) independent experiments are indicated.

#### Role of bile salts in the diversification process

Our previous work has shown that *ompB* mutants entirely outcompeted the ancestral strain during *in vitro* competition in the presence of bile salts, suggesting that their selection *in vivo* in the gut was driven by the presence of bile salts [15]. Further investigation of the bile salts resistance phenotype revealed that *ompB^SG1^* mutant was not affected by concentration of 0.8% of bile salts (cholic acid: deoxycholic acid[1∶1]) whereas the ancestral bacteria displayed a slower growth rate (Figure S9). In contrast, neither *flhDC* nor *malT* deletion conferred any fitness gain in the presence of bile salts (Figure 3C and Figure S9).

In chemostat with bile salts, the *ompB^SG1^* mutation conferred a fitness gain only during the first hours following inoculation, when the carrying capacity of the culture had not yet been reached (Figure 3C). To elucidate the lower fitness advantage of *ompB^SG1^* strain in the presence of bile salts in continuous cultures as opposed to batch cultures, bile salts were added to a continuous culture initiated with WT bacteria that had already reached the maximal carrying capacity of the chemostat culture (12 hours after inoculation). In these conditions, the presence of bile salts only slightly inhibited bacterial growth, probably due to the general stress response induced by the slow growth and the resulting repression of the OmpF porin [48], [49].

**Figure 4:**
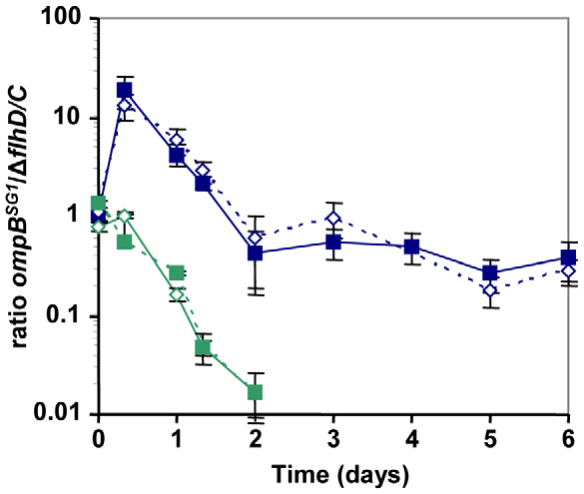
Effect of bile salts on the competition between ompBSG1 and ▵flhDC mutants. Evolution over time (in days) of the ratio of *ompB^SG1^* (possessing the SG1 *envZ* mutation) to ▵*flhDC* mutants in chemostats, in LB (green symbols) or in LB supplemented with bile salts (blue symbols). The strains were differentiated either by fluorescent markers (plain lines) or by their motility phenotypes in motility plates (dashed lines). In the absence of bile salts, the ▵*flhDC* strain rapidly excluded the competitor, while in the presence of 0.8% bile salts an equilibrium was observed after a few days.

Interestingly, after one day of competition between the WT and the *ompB^SG1^* strains, non-motile mutants were selected in the WT population, and an equilibrium was obtained after a few days between the initially inoculated *ompB^SG1^* bacteria and the *de novo* selected non-motile bacteria (Figure 3D). Accordingly, following inoculation of a chemostat containinf bile salts by Δ*flhD* and *ompB^SG1^* mutated bacteria in equal proportions, the two strains coexisted over several days (Figure 4). These results suggest that the distinctive competitive ability of the two mutants, respectively improved growth in nutrient limited conditions and bile salts resistance, associated with a trade-off between growth ability and stress resistance, are sufficient to maintain a balance between the two strains.

### *In vitro* selection of the gut-selected mutations

**Figure 5:**
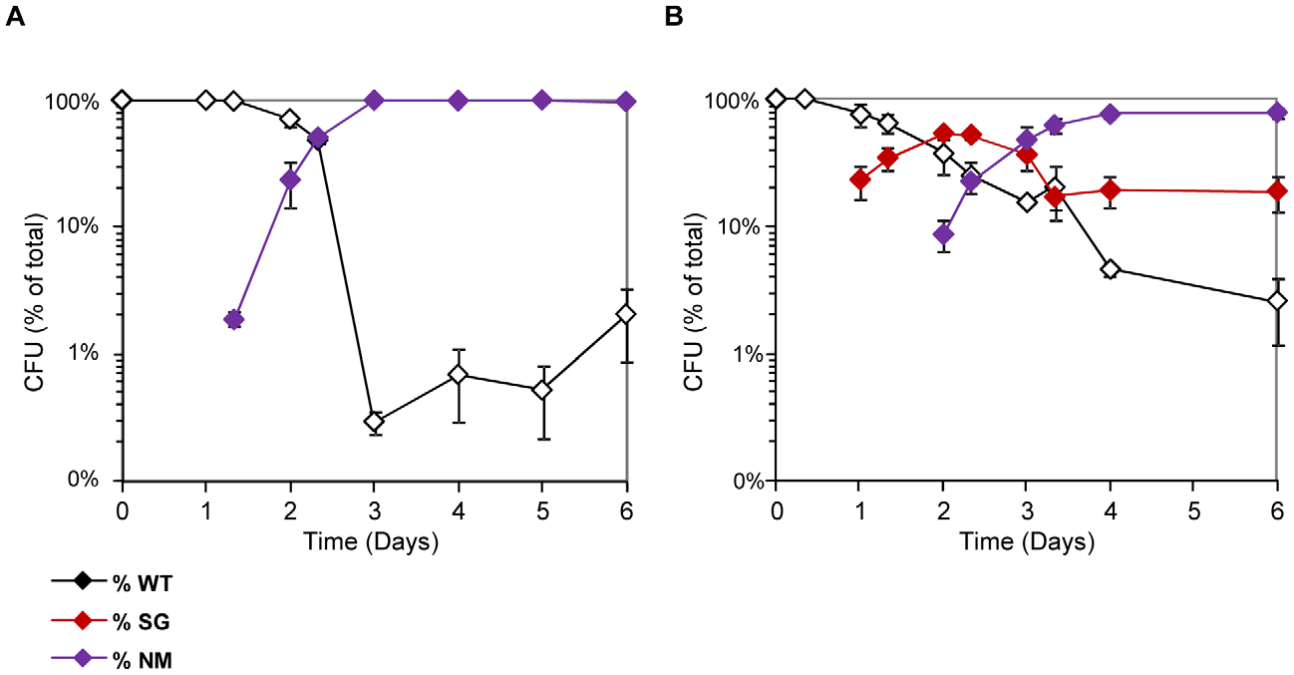
NM and SG morphotypes are selected in continuous cultures supplemented with bile salts. (A) Evolution over time (in days) of the CFU of motility morphotypes following inoculation of chemostats with the WT MG1655 strain. Non-motile (NM) variants are rapidly selected. (B) The same evolution is observed when bile salts are added to the chemostat culture medium at 0.8% w/v. In addition to the non-motile variants, small granulous colonies (SG) are selected.

The above results suggested that *in vitro* conditions could be defined that reproduced the equilibrium between the two mutants selected *in vivo* during gut colonization. To demonstrate that adaptive radiation can stem from the acquisition of distinct capabilities to resist stress, we inoculated continuous cultures only with WT bacteria and followed the appearance of new phenotypes. Non-motile mutants were rapidly selected following inoculation (Figure 5A), as already observed during the *in vitro* competition experiments described above. The *flhDC* genomic region could not be amplified by PCR, indicating that this genomic region was deleted in the mutants. In batch cultures however, non-motile mutants were not selected, in line with the fact that the advantage conferred by *flhDC* deletion is observed only in nutrient-limited conditions.

The selection process was different when bile salts were added into the continuous culture tubes. Strikingly, bacteria forming small granulous colonies on motility plates were selected in the first place, similar to the SG colonies arising during *in vivo* selection (Figure 5B). Sequencing of the *ompB* operon of four granulous clones revealed that they each possessed a different point mutation in *envZ* (Table 4), similarly to the granulous colonies-forming mutants selected in the gut. Two of the identified mutations have been described previously are known to promote OmpR phosphorylation [50]. One day after selection of *ompB* mutants in the continuous cultures, non-motile mutants rapidly invaded the microbial populations. Thus, both the nature and the relative timing of the mutations selected in the gut can be reproduced in continuous cultures containing bile salts. Both *in vitro* and *in vivo*, late selection of *flhDC* bacteria is consistent with the improved advantage of these mutants in slow growth conditions. *In vivo*, we verified that no change over time in bile acid concentration could explain the change in frequency of *ompB* mutants (Figure S10). Moreover, as observed in the gut, once selected the two types of mutants coexisted until the end of the experiment.

**Table 4:** Mutations in ompB selected in chemostats with bile salts.

*envZ* residue change	Phenotype on TM plate	Motility phenotype
**Q228P**	pink	granulous
**P248Q** *	pink	granulous
**T119K**	pink	granulous
**Q283P** *	pink	granulous

*Mutation described in [50].

A mutation in *envZ*, the first gene of the *ompB* operon, was found on each granulous mutant selected in chemostat in the presence of bile salts.

That mutants selected in the mouse gut are also selected in anaerobic continuous cultures containing bile salts strongly suggests that these environmental factors drive the selection of *ompB* and Δ*flhDC* mutants in the gut. It also confirms that OmpR regulation is a general and very efficient mechanism of bile salt resistance in *E. coli* and a key player in *E. coli* adaptation to the mammalian intestinal environment.

## Discussion

Herein we show that upon colonization of an initially germ-free mouse gut by an *E. coli* strain, three types of mutants are systematically selected which then stably coexist, a situation defining adaptive radiation. The first selected mutants show increased resistance to bile salts due to a modification in regulation by OmpR. Mutants possessing a null mutation in the positive regulator of the maltose regulon, *malT*, are concomitantly selected. Then, mutants with deletions in the *flhDC* operon are selected, due to their improved growth in the very densely populated gut conditions. Diversification of *ompB* and *flhDC* mutants could be reproduced in an *in vitro* continuous flow environment mimicking ecological parameters in the mouse distal gut.

A common cause of diversification is resource specialisation of strains whose improved capacity to exploit one resource is associated with decreased capacity to exploit other resources [5]. In such a scenario, competition is avoided because organisms partition resources by expressing specific substrate preferences, by spatial distribution and/or by cross-feeding (one species excreting a substrate used by another one). This mechanism, demonstrated in controlled environments, is suggested to contribute to the high diversity of strains *in vivo* in the intestine. We could not demonstrate in our study any distinct spatial distribution of the motile versus non motile mutants *in vivo* in the gut. Furthermore the selection of non motile mutants was also observed in the unstructured environment of chemostats. A role of cross-feeding in driving diversification was also unlikely as the selection of *ompB* and *flhDC* mutants was observed both *in vitro* in LB with bile salts and *in vivo* in the gut, two different nutritional environments in which by-products of *E. coli* metabolism are most probably different.

Rather, we observed that the selection and cohabitation of *ompB* and *flhDC* mutants stemmed from a trade-off between bile resistance and nutritional competence in the very crowded gut environment. Fitness trade-offs are known to be crucial if diversity is to be maintained [35]. Such a trade-off can be generated if an external factor, such as the host, limits the growth of the most efficient strains. The host immune system is thought to play a preponderant role in this respect [5], [51]. Thus, host IgA response was shown to drive the evolution of *Bacteroides thetaiotaomicron* in a gnotobiotic mouse model [22]. Here the very early selection of *ompB* mutants, detected after only one day of colonization, precluded this mechanism. Downmodulation of flagellin by the *ompB* and *flhDC* mutants may allow *E. coli* to circumvent host innate inflammatory responses, notably elicited via binding of flagellin to epithelial TLR5. Yet, no significant induction of inflammatory signals could be detected in WT mouse ileum even at very early time points (Figure S5) and the same selection of *ompB* and *flhDC* mutants was observed in MyD88−/− mice that cannot signal via TLR5 (Figure S4). Moreover a comparable selection of *ompB* and *flhDC* mutants was observed in chemostat, eliminating the role of host proinflammatory responses and rather pointing to the key role of bile salts in selecting *ompB* mutants and initiating adaptive radiation in the gut environment of monoxenic mice.

Bile salts, contained in the bile digestive secretion, emulsify and solubilize lipids. These physico-chemical properties are central to their function in the absorption of lipids in the distal small intestine but also give them bactericidal capabilities [52], [53]. This effect is reminiscent of the microbicidal properties of defensins which are important actors of the gut innate immune responses and act by permeabilizing bacterial membranes [54]. Selection of *ompB* mutants in response to bile salts may thus be considered as a new example of diversification driven by the host innate immune response. Starvation increases resistance to bile salts, probably via repression of the outer membrane porin OmpF, involved in bile salts resistance [53]. The selected point mutations in *ompB* lead to an increased phosphorylation of OmpR and result in lower outer membrane permeability. As a consequence, *E. coli* becomes constitutively resistant to bile salts even during rapid growth in rich environments, explaining their very large fitness gain during the first two days of colonization. Another consequence of the selected *ompB* mutations is to improve the resistance to β-lactam antibiotics (Figure S11 and [55]). This indirect effect of bile salts exposure has to be taken into account to evaluate the *in vivo* sensitivity to antibiotics.

Interestingly, once the bacterial population has reached its maximal size, either *in vivo* or in chemostat cultures, *flhDC* mutants were selected in the populations that maintained the ancestral *ompB* allele. Since starvation increases resistance of bile salts, the fitness advantage conferred by the *ompB* mutation decreased during slow growth, allowing the selection of other mutants. Our results demonstrate that *flhDC* mutants present an important growth advantage and reach a higher biomass than the ancestral strain in chemostat cultures, likely explaining their selection at high bacterial density. This advantage might be linked to the gain of energy devoted to flagella production and rotation. Flagella expression is known to be high during slow growth, either when the culture enters stationary phase, or during growth on poor carbon sources [56], [57], [58]. Liu et al. proposed that high motility in limited nutrients availability reflects a strategy known as risk-prone foraging whereby bacteria take a risk and use the flagellar system to actively search out for better conditions [58]. Here we show that, in the gut and in continuous cultures, flagella expression is also very high. The high cost of flagella expression might promote the *in vivo* selection of mutants with low or no expression of flagellin and thus explain why 50% of *E. coli* natural isolates are either non-motile or very poorly motile, despite the clear advantage of motility in some conditions [30]. However we cannot rule out that the advantage of the *flhDC* deletion is partly due to another mechanism, as numerous genes that are not directly involved in flagellar structure and motor function are downregulated two-fold or more in a MG1655 *flhDC* deletion mutant strain [57].

*MalT* mutants were not selected in chemostats, indicating that the selective pressure driving their selection is specific to the gut and has yet to be discovered. Interestingly, selection of Mal^−^ bacteria has been repeatedly observed following colonization of streptomycin treated mice by the *E. coli* pathogenic strain 536 (Diard M., personal communication), suggesting an advantage of repressing the maltose regulon in certain conditions in the gut. Compared to other sugar-utilizing pathways, the maltose system is exceptional in two ways. First, the porin LamB is not only crucial for maltose and maltodextrines uptake, it has also a more general role in outer membrane permeability and carbohydrate uptake during growth at low extracellular sugar concentrations [59]. Interestingly, high expression of the maltose regulon is known to be deleterious at high growth rates in glucose limited chemostats [60] and in populations of *E. coli* evolved in the Lenski's Long Term Evolution Experiment, in which cultures alternate phases of rapid and slow growth in a glucose-limited medium [61]. In the gut of monoxenic animals repression of the LamB porin may perhaps also confer increased resistance to high osmolarity.

It has been proposed that stress is an important source of strain variation in bacteria. Indeed, the two main mechanisms of stress response (reduction of the porin-mediated outer membrane permeability and the RpoS-controlled General Stress Response) both involve a trade-off between resistance to stress and resource uptake, called Self-Preservation And Nutritional Competence (SPANC) balance [62]. Here, we show that the SPANC balance not only allows the selection of different variants, but also enable their coexistence, as selected *ompB*, *malT* and *ΔflhDC* mutants coexist in the mouse gut until the end of the experiment, illustrating adaptive radiation.

The role of stress as a driving diversification force has been described by mathematical modelling essentially in the case of allelopathy (one competitor produces a toxin detrimental only to the other species) or in the case of a resistant competitor removing the stress from the environment [63], [64]. In the present situation, the production of bile salts by the host is most probably independent of the proportion of each bacterial mutant. Moreover, we have verified that the resistant competitor is not able to modify or degrade bile salts in the gut. However, we cannot exclude that the coexistence of mutants is driven by frequency-dependent phenomena, as bile salt concentration is variable with time in the gut, bile secretion following the ingestion of meals. Accordingly, bile salts concentration was slightly variable in our chemostat system, as fresh medium was refilled only once or twice a day, and bile salts toxicity declined between medium refilling (data not shown). It is thus possible that *ompB* and *flhDC* mutants are favoured during high and low bile acids concentrations respectively, leading to an overall equilibrium. Yet we did not observe any evidence of daily oscillations in the frequency of the various mutants in the gut or in the chemostats (Figure S1). Alternatively, the observed diversification might simply result from a trade-off between self-preservation and nutritional competence (SPANC trade-off). Indeed recent work suggests that SPANC trade-off alone might be sufficient to drive diversification [65], [66]. Using a mathematical model assuming that resistance to stress is negatively correlated to nutritional competence, the authors showed that equilibrium population could support two or more mutants affected in their levels of stress resistance. They further suggested that genetic variability during extraintestinal infections by *E. coli* might result from distinct levels of RpoS expression [66]. Yet, our results argue against a role of Rpos in our model. Notably the increased yield of Δ*flhDC* mutants in chemostats was not dependent on *rpos* gene.

In conclusion, our study demonstrates that the presence of a stress which competitors have different ability to deal with, associated with a trade-off in terms of nutritional competence, represent necessary and sufficient conditions to select for and maintain several mutants. In the gut, strains with diminished nutritional competence can be selected for if they possess specific resistance mechanisms to a stress imposed by the host. The selective pressure exerted by bile salts highlights a novel role of host innate defense mechanisms in driving adaptive radiation in the gut.

## Materials and Methods

Animal experiments procedures were carried out in accordance with the European guidelines for the care and use of laboratory animals.

### Bacterial strains

All strains were derived from the commensal *E. coli* K12 MG1655 sequenced strain [67]. The reporter strain MG1655 p*fliC*-YFP P2*rrnB*-CFP strain used to monitor activity of *fliC* promoter and the MG1655 ptet-GFP *ompB^SG1^-cat* (containing the SG1 mutant *ompB* allele) have been described elsewhere [15]. *flhDC::Kan*, *malT::Kan* and *rpoS::Kan* deletions were introduced by P1 transduction from the KEIO collection strains (Baba et al, 2006) into the MG1655 ptet-GFP strain.

### Measure of growth rate in defined medium

M9 minimal medium was supplemented with D-glucose (0.2%, wt/wt), N-acetyl-D-glucosamine (0.2%, wt/wt), L-arabinose (0.2%, wt/wt), L-fucose (0.2%, wt/wt), D-galactose (0.2%, wt/wt), D-gluconate (0.2%, wt/wt), D-glucuronate (0.2%, wt/wt), lactose (0.2%, wt/wt), maltose (0.2%, wt/wt) or D-mannose (0.2%, wt/wt). Pre-cultures (10 ml) were grown overnight at 37°C with shaking in 50 ml tubes in M9 minimal medium with sugar (0.2%, wt/wt). These cultures were then washed three times in M9 minimal media, and diluted 50 fold in M9 minimal media containing the same carbon source. Growth was monitored spectrophotometrically every 5 minutes in 96 wells microplates with a microplate reader (iEMS, Labsystem). Generation times were calculated during exponential phase (absorbance at 600 nm around 0.05) over a one hour period from three independent experiments.

### Chemostat culture and sampling

The chemostats used in these experiments were set up with modifications as described in [68]. Briefly, chemostat vessels (tubes) kept at 37°C in a dry bath were controlled by two peristaltic pumps, one which maintained a constant flow of fresh media and one simultaneously removing the waste at the same rate. Chemostat cultures were agitated and aerated by bubbling filter-sterilized nitrogen (nitrogen 4.5, Linde Gas). Samples of 500 µl were taken directly from the chemostat vessel.

### Bacterial counts and motility

Bacterial motility was monitored in soft agar plates (4 g/L Agar in Luria broth medium (LB)) at 37°C for 16 h. The ability to use maltose was monitored in tetrazolium maltose (TM) indicator plates, as Mal+ and Mal- bacteria form white and red colonies respectively when spread on these plates. TM media is composed of Tryptone (10 g/L, Becton Dickinson, MD, USA), yeast extract (1 g/L, Becton Dickinson, MD, USA), NaCl (5 g/L), Agar (16 g/L), maltose (5 g/L, Acros Organics, New Jersey, USA) and tetrazolium dye (50 mg/L, Sigma).

### Confocal microscopy

For detecting YFP expression (*pfliC-yfp*) *in situ*, ileal, cecal and colonic tissues were fixed in paraformaldehyde (4% in PBS, pH 7.4 overnight, 4°C), washed with PBS, equilibrated in PBS (20% sucrose, 0.1% NaN3 overnight, 4°C), embedded in OCT (Sakura, Torrance, CA), snap-frozen in liquid nitrogen and stored at −80°C. Cryosections (7 µm) were air-dried for 2 h at room temperature and fixed in cold acetone (15 min). DNA was stained with PI (Propidium iodide, 0.5 mg ml-1; Sigma). F-Actin was visualized by staining with Alexa-647-conjugated phalloidin, as indicated (Molecular Probes). Sections were mounted with Vectashield hard set (Vector laboratories) and sealed with nail polish. Images were obtained using a Leica confocal microscope (Leica).

### FliC expression assays

Samples of *E. coli* MG1655 p*fliC-yfp* P2*rrnB-cfp* cultures coming either from exponentially growing cultures in LB (OD at 600 nm between 0.15 and 0.2), from 24 h stationary phase cultures, from chemostat cultures or from mice feces were spread on agarose for imaging with a camera CoolSNAP HQ (Princeton Instruments) at 63× magnification by a microscope (Zeiss 200 M; Zeiss), in phase contrast and in fluorescence at wavelength 514 nm (YFP) and 420 nm (CFP) during 1 s exposure time. Excitation light was limited to 50% of the output of the 100-W Hg vapor lamp. Images were treated with the Metamorph software (Universal Imaging). Image analysis procedure identified cells and then quantified their mean fluorescent intensities with YFP and CFP filter sets. We analysed more than 1,000 cells for each condition. Fluorescent background of the agarose media was subtracted from each value of fluorescence. p*fliC* expression was expressed as the ratio of YFP to CFP fluorescence, since protein production under the control of the promotor P2 of the ribosomal gene *rrnB* is steady and independent of bacterial growth rate [69], [70], [71].

### Sequences

Sequencing of the *ompB* locus and the *malT* gene was carried out on purified PCR amplification products using standard procedures at Institut Cochin sequence facilities.

### Bacterial competition

#### *In vivo* competitions

MG1655 ptet-GFP *ompB^SG1^-cat* and MG1655 ptet-RFP *E. coli* were grown in LB for 16 h and mixed at the 1∶1, 1∶100, 1∶1000 SG to WT ratios. Mutant and WT population sizes were determined every 12 hours by counting red and green fluorescent CFU on plates containing 50 µM anhydrotetracycline (Acros Organics) during 5 days following colonization.

#### *In vitro* competitions

Strains were grown in LB for 16 h. 100 µL of the pre-culture of the mutant and of the reference parental strain were inoculated in 15 mL of LB or LB supplemented with bile salts (Bile salts N°3, Difco) at 0.8% (M/W), either in chemostats or in 50 ml tubes for high growth rate as specified. For competition in 50 ml tubes, the OD600 of the culture was maintained below 0.15 by regular dilution. Mutant and parental population sizes were determined regularly by counting SG and LS populations on motility plates, or red, pink or white colonies on TM plates as described above. The selective advantage of *flhDC* mutant compared to WT strain was estimated by fitting an exponential curve to the evolution of the *flhDC*/WT ratio.

### Mice and *in vivo* colonization experiments

Conventional and germ-free WT C3H/HeN mice were bred at the ANAXEM facilities, INRA, Jouy-en-Josas, France. MyD88−/− C57BL/6N mice come from the CDTA, Orleans, France. They were decontaminated by adding an antibiotics cocktail (Ampicillin sodium salt, 1 g/L, Sigma; Vancomycin, 500 mg/L, Sigma; Neomycin sulphate, 1 g/L, Sigma; Metronidazole, 1 g/L, Sigma) to the drinking water for three weeks. Sterility was checked by examination of feces samples by microscopy and cultures.

Germ-free and gnotobiotic mice were reared in isolators (Ingenia, Vitry/Seine, France) in individual cages. They were fed ad libitum on a commercial diet sterilized by gamma irradiation (40 kGy) and supplied with sterile water. For colonization experiments, 8–12 week-old mice were inoculated per os with 10^5^ bacteria from the *E. coli* MG1655 p*fliC-yfp* P2*rrnB-cfp* strain in 0.5 mL of 10 mM MgSO4 solution. Colonization was monitored by bacterial counts in individual freshly harvested fecal samples as described [72].

#### Depletion of gut bacteria in MyD88^−/−^ mice

In conventional C57BL/6 MyD88^−/−^ mice, the microbiota was eliminated as described in [21]. Briefly, mice were provided ampicilin (1 g/L, Sigma), vancomycin (500 mg/L, Sigma), neomycin sulphate (1 g/L, Sigma) and metronidazole (1 g/L, Sigma) in drinking water for three weeks prior to colonization with the *E. coli* MG1655 strain. Animal experiments were carried out in accordance with the European guidelines for the care and use of laboratory animals.

### Quantitation of gene expression with real-time PCR

#### Bacterial genes

Total RNA was extracted from 5 ml of bacterial culture growing in chemostat using the RNeasy kit (Qiagen), according to the manufacturer's instructions. RNA was treated with four units of the Turbo DNA-free (Ambion) for 1 h at 37°C. RNA integrity was determined by Agilent 2100 Bioanalyzer (Agilent Technologies). The cDNA synthesis was performed using 2 µg RNA with random hexamers (12.5 ng/ml) and the Superscript II RNAse H_ kit 5 (invitrogen) according to the manufacturer's instructions. The real-time PCR experiments were performed using the SYBRgreen PCR Master Mix (Applied Biosystems) and specific primers (Table S1). The *rpoD* gene was chosen as a reference gene for ΔCt calculation. Amplification and detection of the specific products were carried out with the 7300 Real Time PCR System (Applied Biosystems).

#### Mouse genes

Ileal tissue was lysed in Trizol (Invitrogen) and total RNA was extracted and cleanup with the RNeasy kit and RNase-free DNase I (QIAGEN) digestion based on the manufacturer's protocol. RNA integrity was determined by Agilent 2100 Bioanalyzer (Agilent Technologies). Two micrograms of total RNA was reverse transcribed to cDNA using random hexamer and M-MLV polymerase (Invitrogen) according to manufacturer's instructions. Quantitative RT-PCR was performed with mouse-specific primers with SYBR-green PCR master mix (Applied Biosystems) (Table S1). cDNA samples were assayed in duplicate and gene expression levels for each sample were normalized relative to Beta actine.

### Bile acid analysis by LC-MS/MS

Total and individual bile acid concentrations were determined by LC-MS/MS analysis in mouse cecal contents.

#### Chemicals and reagents

CA, DCA, CDCA, UDCA, LCA, HCA and corresponding glycine and taurine conjugates were purchased from Sigma-Aldrich, Saint Quentin Fallavier, France; 3-sulfate conjugates were a generous gift from J Goto, Niigita University of Pharmacy and Applied Life Science, Niigata, Japan; 23-nor-5β-cholanoic acid-3α,12β diol, muricholic acid derivatives, their glycine and taurine conjugates were purchased from Steraloids Inc, Newport, RI. Acetic acid, ammonium carbonate and ammonium acetate were from Sigma-Aldrich. Standard solutions: 1 mg/ml stock solutions were prepared in methanol and stored at −20°C. The stock solutions were pooled and diluted to obtain mixed calibration solutions of 31.3 µg/ml to 31.3 ng/ml of each bile acid.

#### Sample extraction

After sampling, around 200 mg of cecal contents were freezed in liquid nitrogen and subsequently freezed-dried; 2 ml of 0.1 M NaOH were added to 0.1 g of dry feces and incubated 1 h at 60°C; 4 ml of distillated water and Norcholic acid (1 mg/ml stock solution in 50% ethanol) was added and the sample was treated 30 s with a Polytron homogenizer (KINEMATICA GMBH) and centrifuged 20 min at 20 000 *g*. The supernatant was discarded and treated as a biological fluid.

Samples of cecal extracts were mixed with 2 µl of internal standard solution (23-nor-5β-cholanoic acid-3α,12β diol; 1 mg/ml). Bile acids were released from proteins by incubation with 0.5 mol/l ammonium carbonate, for 30 min at 60°C. Sample clean-up was performed by centrifugation (4000 *g* for 10 min) and solid-phase extraction, using reverse phase Chromabond C_18_ cartridges (100 mg) (Macherey-Nagel). Solid-phase extraction was processed on a vacuum manifold. The cartridge was rinsed successively with water (20 ml), hexane (10 ml) to discard neutral lipids and again with water (20 ml). Bile acids were eluted with methanol, dried by evaporation under a nitrogen stream at 50°C and dissolved in 150 µl methanol. 5 µl were injected into the LC-MS/MS system.

#### LC-MS/MS conditions

Chromatographic separation was performed using HPLC Agilent 1100 fitted with the analytical column Restek C18 Pinnacle II (250 mm×3.2 mm 5 µm; Restek). Separation was achieved at a flow rate of 0.3 to 0.5 ml/min. The mobile phase A was ammonium acetate 15 mM, pH 5.3, and solvent B was methanol. HPLC was in series with the turbo ion spray source of the mass spectrometer QTRAP 2000 (Applied Biosystems-SCIEX). Electro-Spray-Ionisation was performed in the negative mode with nitrogen as the nebulizer gas. Nebulizer, curtain and heater gas were set at 40, 20 and 40, respectively. The temperature of the evaporation gas was set at 400°C. The ion spray, declustering and entrance potentials were set at −4500 V, −60 V and −10 V, respectively. The MS/MS detection was operated with a unit resolution in MRM mode. The dwell time for each transition was set at 70 ms. Data were acquired with Analyst software, Version 1.4.2. Multiple Reaction Monitoring was performed by examination of the transition reactions from precursor ions to product ions after collision induced dissociation of the taurine or glycine residue. For unconjugated mono-, di- and tri-hydroxylated bile acids, *m*/*z* 375, 391 and 407 respectively, were selected as precursor and product ions. For the internal standard (23-nor-5β-cholanoic acid-3α,12β diol), *m/z* 377 was selected. For glycine conjugates, *m*/*z* 432, 448 and 464 representing mono-, di- and tri-hydroxylated bile acids, respectively, were selected as precursor ions and *m*/*z* 74, as product ion. For taurine conjugates, *m*/*z* 482, 498 and 514 corresponding to mono-, di- and tri-hydroxylated bile acids, respectively, were selected as precursor ions, and *m*/*z* 80, as the product ion. For sulfo conjugates, *m*/*z* 97 was selected as the product ion.

## Supporting Information

Figure S1 Selective advantage of *envZ* mutation decreases after day 3. Evolution over time (in days) of the ratio of *envZ* mutant to WT CFU in the feces of mice inoculated with MG1655 ptet-GFP *ompBSG1-cat* (containing the SG1 *envZ* mutation) and MG1655 ptet-RFP *ompB-cat* (containing the WT *envZ* allele) mixed at initial ratios of 1∶1 (diamonds), 1∶100 (squares), and 1∶1,000 (triangles). The error bars represent the standard error of the mean of four mice.
(PPT)

Figure S2 SG1 mutant displays reduced expression of lamB. qRT-PCR on bacterial cultures demonstrate difference between the Ct of the *lamB* gene and the Ct of the endogenous reference gene (*rpoD*) in WT and SG1 strains.
(PPT)

Figure S3 Radiative evolution in individual mice. Evolution over time (in days) of CFU morphotypes in the feces of 12 mouse inoculated with the *E. coli* MG1655 p*fliC*-*yfp* strain. The percentages of colony phenotypes in motility agar are indicated: large smooth (LS, similar to the ancestor), small granulous (SG) and totally non motile (NM). In 8 mouse, colonies phenotypes were also observed on tetrazolium maltose plates, and the percentage of red colonies corresponding to bacteria unable to use maltose (Mal^−^) is indicated (dotted red lines).
(PPT)

Figure S4 The same diversification is observed in MyD88^−/−^ and in WT mice. Evolution over time (in days) of new morphotypes CFU (mean +/− standard error of the mean) in the feces of mice inoculated with the *E. coli* MG1655 strain. No significant difference is observed between the WT mice (continuous lines) and MyD88^−/−^ mice (dotted lines).
(PPT)

Figure S5 *E. coli* MG1655 fails to induce any detectable proinflammatory response *in vivo* upon intestinal colonization of germ-free mice. Relative mRNA expression of CCL-20 and IL-12p40, two genes induced after binding of flagellin to its cognate receptor TLR5, in germ-free mice (GF, n = 7), conventional mice (Cv, n = 7) and in mice monocolonized with *E. coli (*n = 6) after 1, 2, 3, 5 or 8 days of colonization. Mean +/− standard error of the mean.
(PPT)

Figure S6 The same proportion of flagellated bacteria is observed in different parts of the gut. A, B and C: images obtained by confocal microscopy of the same cecal area illustrating the homogenous repartition of flagellated bacteria: (A) propidium iodide (PI) staining, (B) Yfp expression from the *pflic-yfp* construct, (C) merge of PI, yfp and alexa647-phalloïdin that marks the brush border. D: percentage of *fliC* expressing bacteria in different areas of the gut: in the caecum and in the colon, and near the epithelium or in the center of the lumen. Samples from two different mice were observed. For each sample, numbers are average of two different positions on two different slides (total of four measures).
(PPT)

Figure S7 Growth of the different mutants. Representative growth curves of the WT and isogenic mutants in 96 wells microplates, either in LB medium (A) or in M9 minimal medium containing glucose (B). 200 µl cultures were covered with oil to prevent evaporation, and were agitated for 1 minute before every reading. Experiments were realized with the plate reader VICTOR Multilabel Plate Reader (PerkinElmer).
(PPT)

Figure S8 Expression profile of E. coli genes known to respond to oxygen availability in aerated cultures, chemosat cultures and in mice caecum. Differences between the Ct of specified genes and the Ct of the endogenous reference gene (*rpoD*) in *E. coli* MG1655 populations growing in different conditions. A: aerated culture (10 ml of culture in 50 ml tubes, OD = 0.6), C: chemostat culture (24 h after inoculation, OD = 0.65) and in mice caecum (M). The product of *cadA* is Lysine decarboxylase, whose expression is increased in anaerobic conditions [73]. *CyoA* and *cydA* code for cytochrome oxidases involved in aerobic and microaerobic respiration respectively. The expression levels of bacterial genes observed in mice is closest to the expression in chemostat than in aerated batch cultures.
(PPT)

Figure S9 The *ompB* mutation of SG1 mutant improve growth in presence of bile salts. Representative growth curves in LB curves of ancestral (WT), one selected mutant of each phenotype (NM1, Mal^−^1 and SG1) and isogenic reconstructed strains supplemented with bile salts (0.5% wt/vol). Plates were incubated at 37°C under continuous orbital agitation in a plate reader (iEMS, Labsystem), and growth was monitored spectrophotometrically at 600 nm every 5 minutes.
(PPT)

Figure S10 Concentration of total bile acids per gram of dry caecal content in germ-free mice prior to colonization (GF) and over time post-colonization with *E. coli* MG1655 (from day 1 (d1) to day 15 (d15)). Individual values (black diamonds) and mean (red dash) are shown. Bile acids were measured by LC-MS/MS.
(PPT)

Figure S11 Changes in antibiotics sensitivity due to the selected *ompB* mutation. Disks containing either 25 mg of Ampicillin (AMP 25) or 10 mg of Streptomycin (S 10) inhibit bacterial growth. The diameter of the inhibition area is bigger for the WT culture than for the *ompB* SG1 mutant. No difference was observed for the other antibiotics tested (chloramphenicol, tetracycline, rifampicine and colibactine).
(PPT)

Table S1 Primer sequences used for qRT-PCR. The table contains the sequence of all primers used for gene expression by semi quantitative RT-PCR.
(DOCX)
