# Supplementary material for: Trade-Off between Bile Resistance and Nutritional Competence Drives Escherichia coli Diversification in the Mouse Gut
Source: PLoS Genet. 2011 Jun 16;7(6):e1002107. doi: 10.1371/journal.pgen.1002107 (PMC3116916; doi:10.1371/journal.pgen.1002107)
Supplement: Table S1 — Primer sequences used for qRT-PCR. The table contains the sequence of all primers used for gene expression by semi quantitative RT-PCR. (DOCX) [file pgen.1002107.s012.docx]

|  | **gene** | **primer name** | **sequence** |
| --- | --- | --- | --- |
| Mice genes | *βactin* | beta actin Fwd | 5'- GCTTCTTTGCAGCTCCTTCGT-3' |
|  |  | beta actin Rev | 5'- ATGCCGGAGCCGTTGTC -3' |
|  | *IL12p40* | IL12p40-fwd | 5’-GGAAGCACGGCAGCAGAATA-3’ |
|  |  | IL12p40-rev | 5’-AACTTGAGGGAGAAGTAGGAATGG-3’ |
|  | *mCCL20* | mCCL20-fwd | 5'-TCTGTGTGCGCTGATCCAA-3' |
|  |  | mCCL20-rev | 5'-TGTGTCCAATTCCATCCCAAA-3' |
| *E. coli* genes | *rpoD* | rpoD-fwd | 5'-GTAGTCGGTGTTCATATCGA-3' |
|  |  | rpoD-rev | 5'-CGTCTGATCATGAAGCTCT-3' |
|  | *lamB* | lamB-fwd | 5′-AACTTCCTCTGGCGGTTGC-3′ |
|  |  | lamB-rev | 5′-ACCTGTCCAACCAATACCGG-3′ |
|  | *cadA* | cadA-fwd | 5'-AGACCGCAGCTACATGGTGAC-3′ |
|  |  | cadA-rev | 5'-CACGCGCTTAGCAATGGTAGC-3′ |
|  | *cyoA* | cyoA-fwd | 5'- AAGTACTGCCCGAACTGGTC-3′ |
|  |  | cyoA-rev | 5'-ACGGTAGCAATGCCCTGT-3′ |
|  | *cydA* | cydA-fwd | 5'-TCTTCGGTTGGGATCGTCTG-3′ |
|  |  | cydA-rev | 5'-ATGCGCTGATACCGAGGATG-3′ |

Supplementary Table S1 :
